# Supplementary material for: How should COVID-19 vaccines be distributed between the Global North and South: a discrete choice experiment in six European countries
Source: eLife. 2022 Oct 18;11:e79819. doi: 10.7554/eLife.79819 (PMC9592081; doi:10.7554/eLife.79819)
Supplement: Supplementary file 1. — (a) Population Census Data for Key Sociodemographic Variables. (b) Country of residence attribute main effect - By subgroups of respondent characteristics (pooled sample). (c) Country of residence attribute: Heterogeneity by respondent’s characteristics (pooled results). (d) Country of residence attribute: Heterogeneity by respondent’s characteristics (German sample). (e) Country of residence attribute: Heterogeneity by respondent’s characteristics (Spanish sample). (f) Country of residence attribute: Heterogeneity by respondent’s characteristics (Italian sample). (g) Country of residence attribute: Heterogeneity by respondent’s characteristics (French sample). (h) Country of residence attribute: Heterogeneity by respondent’s characteristics (Polish sample). (i) Country of residence attribute: Heterogeneity by respondent’s characteristics (Swedish sample). (j) Country of residence attribute main effect - By subgroups of regional case incidence (pooled sample). (k) Country of residence attribute: Heterogeneity by regional case incidence (pooled results). (l) Country level differences in case incidence, vaccination rates, willingness and threat perception [file elife-79819-supp1.docx]

**Supplementary File 1**

**Supplementary File 1a** - Population Census Data for Key Sociodemographic Variables

| **Country** | **female** | **18-24 years** | **25-34 years** | **35-44 years** | **45-54 years** | **55-64 years** | **65+ years** | **low education** | **medium education** | **high education** |
| --- | --- | --- | --- | --- | --- | --- | --- | --- | --- | --- |
| France | 51.67% | 8.09% | 11.6% | 12.4% | 13.3% | 12.6% | 20.4% | 22.3% | 42.4% | 35.3% |
| Germany | 50.66% | 7.41% | 12.7% | 12.3% | 14.4% | 14.9% | 21.8% | 20.1% | 52.7% | 27.2% |
| Italy | 51.29% | 6.91% | 10.7% | 13.0% | 16.1% | 14.1% | 23.2% | 39.0% | 43.1% | 17.9% |
| Poland | 51.60% | 7.26% | 14.2% | 16.1% | 12.6% | 13.4% | 18.2% | 12.9% | 58.1% | 28.9% |
| Spain | 50.99% | 7.54% | 10.2% | 15.4% | 15.9% | 13.3% | 19.6% | 38.2% | 25.8% | 36.0% |
| Sweden | 49.69% | 7.89% | 14.1% | 12.4% | 13.0% | 11.5% | 20.0% | 20.7% | 41.1% | 38.3% |

**Supplementary File 1b** – Country of residence attribute main effect - By subgroups of respondent characteristics (pooled sample)

|  | (1) | (2) | (3) | (4) | (5) | (6) | (7) | (8) | (9) | (10) | (11) | (12) | (13) |
| --- | --- | --- | --- | --- | --- | --- | --- | --- | --- | --- | --- | --- | --- |
|  | Full sample | Male | Female | <45 yrs. | >45 yrs. | Low edu | High edu | Not high-risk | High-risk | Low threat | High threat | Unemployed | Employed |
| **Country of residence** |  |  |  |  |  |  |  |  |  |  |  |  |  |
| Respondents’ country | Reference category | | | | | | | | | | | | |
| Global South | 1.16∗∗∗  [1.10,1.23] | 1.03  [0.96,1.11] | 1.33∗∗∗  [1.23,1.44] | 1.32∗∗∗  [1.23,1.41] | 0.96  [0.88,1.06] | 0.90∗  [0.82,0.99] | 1.35∗∗∗  [1.26,1.44] | 1.28∗∗∗  [1.20,1.37] | 0.98  [0.89,1.07] | 1.15∗∗∗  [1.06,1.24] | 1.18∗∗∗  [1.09,1.28] | 1.19∗∗∗  [1.08,1.32] | 1.15∗∗∗  [1.08,1.22] |
| Pseudo *R*^2^ | 0.16 | 0.13 | 0.20 | 0.16 | 0.17 | 0.15 | 0.17 | 0.17 | 0.16 | 0.14 | 0.18 | 0.18 | 0.16 |
| Observations | 96480 | 48448 | 47648 | 57280 | 38816 | 34896 | 61200 | 61456 | 34640 | 47808 | 48224 | 30128 | 65968 |

***Notes:*** *Outcome: Choosing the respective candidate to receive the vaccine. Coefficients are odds ratios based on conditional logit estimations (respondent-level fixed effects) with standard errors clustered at the respondent level. Estimations were conducted with all four attributes, but only the results for the country of residence attribute are shown here. Columns 2-13 represent the exact coefficients shown in Figure 1 in the main body of the paper. Results to be interpreted relative to the indicated reference category, i.e. in the case of country of residence, relative to the preference for the vaccine being given to a person living in the country of the survey respondent answering the question. 95% confidence intervals in brackets. ^∗^ p < 0.05, ^∗∗^ p < 0.01, ^∗∗∗^ p < 0.001.*

**Supplementary File 1c** – Country of residence attribute: Heterogeneity by respondent’s characteristics

(pooled results)

|  | (1) | (2) | (3) | (4) | (5) | (6) | (7) |
| --- | --- | --- | --- | --- | --- | --- | --- |
| ***Country of Residence*** |  |  |  |  |  |  |  |
| Respondents’ Country | Reference Category | | | | | | |
| Global South | 1.16^∗∗∗^ | 1.05 | 1.36^∗∗∗^ | 0.85^∗∗∗^ | 1.32^∗∗∗^ | 1.11^∗∗^ | 1.26^∗∗∗^ |
|  | [1.10,1.23] | [0.97,1.14] | [1.27,1.45] | [0.78,0.93] | [1.24,1.41] | [1.03,1.19] | [1.14,1.38] |
| Global South × Female respondent |  | 1.22^∗∗∗^ [1.10,1.36] |  |  |  |  |  |
| Global South × Respondent ≥ 45 |  |  | 0.68^∗∗∗^ [0.61,0.76] |  |  |  |  |
| Global South × Higher educated respondent |  |  |  | 1.63^∗∗∗^ [1.46,1.82] |  |  |  |
| Global South × High-risk respondent |  |  |  |  | 0.70^∗∗∗^ [0.63,0.78] |  |  |
| Global South × High perceived threat respondent |  |  |  |  |  | 1.10 |  |
|  |  |  |  |  |  | [0.99,1.22] |  |
| Global South × Employed respondent |  |  |  |  |  |  | 0.89^∗^ [0.80,1.00] |
| Log likelihood | -47779.22 | -47551.104 | -47485.96 | -47434.20 | -47501.49 | -47538.99 | -47569.78 |
| AIC | 95576.44 | 95122.21 | 94991.91 | 94888.40 | 95022.98 | 95097.99 | 95159.56 |
| BIC | 95661.73 | 95216.94 | 95086.64 | 94983.13 | 95117.71 | 95192.71 | 95254.29 |
| Pseudo R^2^ | 0.16 | 0.16 | 0.16 | 0.17 | 0.16 | 0.16 | 0.16 |
| **Observations** | **96480** | **96096** | **96096** | **96096** | **96096** | **96032** | **96096** |

***Notes:*** *Outcome: Choosing the respective candidate to receive the vaccine. Coefficients are odds ratios based on conditional logit estimations (respondent-level fixed effects) with standard errors clustered at the respondent level. Estimations were conducted with controlling for the main effects of the other three attributes, but only the results for the country of residence attribute are shown here. Columns 2-7 indicate the degree of statistical (in-)significance of the subgroup differences presented in Figure 1 in the main body of the paper and Table S2 of the supplementary material. Results to be interpreted relative to the indicated reference category, i.e. in the case of country of residence, relative to the preference for the vaccine being given to a person living in the country of the survey respondent answering the question. 95% confidence intervals in brackets. ^∗^ p < 0.05, ^∗∗^ p < 0.01, ^∗∗∗^p < 0.001.*

**Supplementary File 1d** – Country of residence attribute: Heterogeneity by respondent’s characteristics (German sample)

|  | (1) | (2) | (3) | (4) | (5) | (6) | (7) |
| --- | --- | --- | --- | --- | --- | --- | --- |
| ***Country of Residence*** |  |  |  |  |  |  |  |
| Respondents’ Country | Reference Category | | | | | | |

| Global South | 0.69^∗∗∗^ | 0.65^∗∗∗^ | 0.89 | 0.57^∗∗∗^ | 0.82^∗∗^ | 0.70^∗∗∗^ | 0.79^∗∗^ |
| --- | --- | --- | --- | --- | --- | --- | --- |
|  | [0.62,0.76] | [0.56,0.75] | [0.77,1.03] | [0.50,0.65] | [0.71,0.94] | [0.61,0.82] | [0.67,0.92] |
| Global South × Female respondent |  | 1.15 |  |  |  |  |  |
|  |  | [0.95,1.40] |  |  |  |  |  |
| Global South × Respondent ≥ 45 |  |  | 0.66^∗∗∗^ [0.54,0.80] |  |  |  |  |
| Global South × Higher educated respondent |  |  |  | 1.70^∗∗∗^ [1.39,2.08] |  |  |  |
| Global South × High-risk respondent |  |  |  |  | 0.73^∗∗^ [0.60,0.88] |  |  |
| Global South × High perceived threat respondent |  |  |  |  |  | 0.96 |  |
|  |  |  |  |  |  | [0.79,1.17] |  |
| Global South × Employed respondent |  |  |  |  |  |  | 0.81^∗^ [0.66,0.99] |
| Pseudo R^2^ | 0.22 | 0.22 | 0.23 | 0.23 | 0.22 | 0.22 | 0.22 |
| Observations | 31424 | 31424 | 31424 | 31424 | 31424 | 31424 | 31424 |

***Notes:*** *Outcome: Choosing the respective candidate to receive the vaccine. Coefficients are odds ratios based on conditional logit estimations (respondent-level fixed effects) with standard errors clustered at the respondent level. Estimations were conducted with controlling for the main effects of the other three attributes, but only the results for the country of residence attribute are shown here. Columns 2-7 indicate the degree of statistical (in-)significance of the subgroup differences presented in Figure 2 in the main body of the paper. Results to be interpreted relative to the indicated reference category, i.e. in the case of country of residence, relative to the preference for the vaccine being given to a person living in the country of the survey respondent answering the question. 95% confidence intervals in brackets. ^∗^ p < 0.05, ^∗∗^ p < 0.01, ^∗∗∗^p < 0.001.*

**Supplementary File 1e** – Country of residence attribute: Heterogeneity by respondent’s characteristics (Spanish sample)

|  | (1) | (2) | (3) | (4) | (5) | (6) | (7) |
| --- | --- | --- | --- | --- | --- | --- | --- |
| ***Country of Residence*** |  |  |  |  |  |  |  |
| Respondents’ Country | Reference Category | | | | | | |

| Global South | 1.79^∗∗∗^ | 1.44^∗∗∗^ | 1.83^∗∗∗^ | 1.49^∗^ | 1.79^∗∗∗^ | 1.47^∗∗∗^ | 2.46^∗∗∗^ |
| --- | --- | --- | --- | --- | --- | --- | --- |
|  | [1.55,2.06] | [1.17,1.76] | [1.57,2.15] | [1.09,2.04] | [1.52,2.10] | [1.20,1.80] | [1.94,3.13] |
| Global South × Female respondent |  | 1.54^∗∗^ [1.18,2.01] |  |  |  |  |  |
| Global South × Respondent ≥ 45 |  |  | 0.91 |  |  |  |  |
|  |  |  | [0.66,1.26] |  |  |  |  |
| Global South × Higher educated respondent |  |  |  | 1.25 |  |  |  |
|  |  |  |  | [0.89,1.76] |  |  |  |
| Global South × High-risk respondent |  |  |  |  | 1.00 |  |  |
|  |  |  |  |  | [0.74,1.37] |  |  |
| Global South × High perceived threat respondent |  |  |  |  |  | 1.45^∗∗^ [1.11,1.90] |  |
| Global South × Employed respondent |  |  |  |  |  |  | 0.63^∗∗^ [0.48,0.84] |
| Pseudo R^2^ | 0.19 | 0.19 | 0.19 | 0.19 | 0.19 | 0.19 | 0.19 |
| Observations | 14800 | 14800 | 14800 | 14800 | 14800 | 14800 | 14800 |

***Notes:*** *Outcome: Choosing the respective candidate to receive the vaccine.* *Coefficients are odds ratios based on conditional logit estimations (respondent-level fixed effects) with standard errors clustered at the respondent level. Estimations were conducted with controlling for the main effects of the other three attributes, but only the results for the country of residence attribute are shown here. Columns 2-7 indicate the degree of statistical (in-)significance of the subgroup differences presented in Figure 2 in the main body of the paper. Results to be interpreted relative to the indicated reference category, i.e., in the case of country of residence, relative to the preference for the vaccine being given to a person living in the country of the survey respondent answering the question. 95% confidence intervals in brackets. ^∗^ p < 0.05, ^∗∗^ p < 0.01, ^∗∗∗^p < 0.001.*

**Supplementary File 1f** – Country of residence attribute: Heterogeneity by respondent’s characteristics (Italian sample)

|  | (1) | (2) | (3) | (4) | (5) | (6) | (7) |
| --- | --- | --- | --- | --- | --- | --- | --- |
| ***Country of Residence*** |  |  |  |  |  |  |  |
| Respondents’ Country | Reference Category | | | | | | |

| Global South | 1.74^∗∗∗^ | 1.56^∗∗∗^ | 1.54^∗∗∗^ | 1.63^∗∗^ | 1.66^∗∗∗^ | 1.71^∗∗∗^ | 2.01^∗∗∗^ |
| --- | --- | --- | --- | --- | --- | --- | --- |
|  | [1.50,2.01] | [1.27,1.91] | [1.30,1.83] | [1.19,2.22] | [1.41,1.96] | [1.38,2.13] | [1.58,2.54] |
| Global South × Female respondent |  | 1.24 |  |  |  |  |  |
|  |  | [0.95,1.64] |  |  |  |  |  |
| Global South × Respondent ≥ 45 |  |  | 1.34^∗^ [1.00,1.78] |  |  |  |  |
| Global South × Higher educated respondent |  |  |  | 1.09 |  |  |  |
|  |  |  |  | [0.77,1.53] |  |  |  |
| Global South × High-risk respondent |  |  |  |  | 1.18 |  |  |
|  |  |  |  |  | [0.86,1.62] |  |  |
| Global South × High perceived threat respondent |  |  |  |  |  | 1.03 |  |
|  |  |  |  |  |  | [0.78,1.36] |  |
| Global South × Employed respondent |  |  |  |  |  |  | 0.80 |
|  |  |  |  |  |  |  | [0.60,1.07] |
| Pseudo R^2^ | 0.10 | 0.10 | 0.10 | 0.10 | 0.10 | 0.10 | 0.10 |
| Observations | 12272 | 11888 | 11888 | 11888 | 11888 | 11888 | 11888 |

***Notes:*** *Outcome: Choosing the respective candidate to receive the vaccine. Coefficients are odds ratios based on conditional logit estimations (respondent-level fixed effects) with standard errors clustered at the respondent level. Estimations were conducted with controlling for the main effects of the other three attributes, but only the results for the country of residence attribute are shown here. Columns 2-7 indicate the degree of statistical (in-)significance of the subgroup differences presented in Figure 2 in the main body of the paper. Results to be interpreted relative to the indicated reference category, i.e. in the case of country of residence, relative to the preference for the vaccine being given to a person living in the country of the survey respondent answering the question. 95% confidence intervals in brackets. ^∗^ p < 0.05, ^∗∗^ p < 0.01, ^∗∗∗^p < 0.001.*

**Supplementary File 1g** – Country of residence attribute: Heterogeneity by respondent’s characteristics (French sample)

|  | (1) | (2) | (3) | (4) | (5) | (6) | (7) |
| --- | --- | --- | --- | --- | --- | --- | --- |
| ***Country of Residence*** |  |  |  |  |  |  |  |
| Respondents’ Country | Reference Category | | | | | | |

| Global South | 1.37^∗∗∗^ | 1.31^∗∗^ | 1.32^∗∗^ | 0.95 | 1.39^∗∗∗^ | 1.22^∗^ | 1.50^∗∗^ |
| --- | --- | --- | --- | --- | --- | --- | --- |
|  | [1.18,1.59] | [1.07,1.59] | [1.10,1.59] | [0.62,1.44] | [1.16,1.65] | [1.01,1.48] | [1.13,1.98] |
| Global South × Female respondent |  | 1.11 |  |  |  |  |  |
|  |  | [0.84,1.47] |  |  |  |  |  |
| Global South × Respondent ≥ 45 |  |  | 1.12 |  |  |  |  |
|  |  |  | [0.84,1.50] |  |  |  |  |
| Global South × Higher educated respondent |  |  |  | 1.52 |  |  |  |
|  |  |  |  | [0.98,2.37] |  |  |  |
| Global South × High-risk respondent |  |  |  |  | 0.97 |  |  |
|  |  |  |  |  | [0.71,1.31] |  |  |
| Global South × High perceived threat respondent |  |  |  |  |  | 1.29 |  |
|  |  |  |  |  |  | [0.97,1.71] |  |
| Global South × Employed respondent |  |  |  |  |  |  | 0.89 |
|  |  |  |  |  |  |  | [0.65,1.23] |
| Pseudo R^2^ | 0.17 | 0.17 | 0.17 | 0.17 | 0.17 | 0.17 | 0.17 |
| Observations | 12256 | 12256 | 12256 | 12256 | 12256 | 12256 | 12256 |

***Notes:*** *Outcome: Choosing the respective candidate to receive the vaccine. Coefficients are odds ratios based on conditional logit estimations (respondent-level fixed effects) with standard errors clustered at the respondent level. Estimations were conducted with controlling for the main effects of the other three attributes, but only the results for the country of residence attribute are shown here. Columns 2-7 indicate the degree of statistical (in-)significance of the subgroup differences presented in Figure 2. Results to be interpreted relative to the indicated reference category, i.e. in the case of country of residence, relative to the preference for the vaccine being given to a person living in the country of the survey respondent answering the question. 95% confidence intervals in brackets. ^∗^ p < 0.05, ^∗∗^ p < 0.01, ^∗∗∗^p < 0.001.*

**Supplementary File 1h**– Country of residence attribute: Heterogeneity by respondent’s characteristics (Polish sample)

|  | (1) | (2) | (3) | (4) | (5) | (6) | (7) |
| --- | --- | --- | --- | --- | --- | --- | --- |
| ***Country of Residence*** |  |  |  |  |  |  |  |
| Respondents’ Country | Reference Category | | | | | | |

| Global South | 0.99 | 0.98 | 1.03 | 1.29 | 0.99 | 1.00 | 1.00 |
| --- | --- | --- | --- | --- | --- | --- | --- |
|  | [0.86,1.15] | [0.79,1.21] | [0.88,1.20] | [0.97,1.71] | [0.83,1.18] | [0.84,1.18] | [0.77,1.30] |
| Global South × Female respondent |  | 1.03 |  |  |  |  |  |
|  |  | [0.78,1.36] |  |  |  |  |  |
| Global South × Respondent ≥ 45 |  |  | 0.86 |  |  |  |  |
|  |  |  | [0.61,1.21] |  |  |  |  |
| Global South × Higher educated respondent |  |  |  | 0.72^∗^ [0.52,0.99] |  |  |  |
| Global South × High-risk respondent |  |  |  |  | 1.01 |  |  |
|  |  |  |  |  | [0.76,1.34] |  |  |
| Global South × High perceived threat respondent |  |  |  |  |  | 0.99 |  |
|  |  |  |  |  |  | [0.74,1.34] |  |
| Global South × Employed respondent |  |  |  |  |  |  | 0.99 |
|  |  |  |  |  |  |  | [0.73,1.34] |
| Pseudo R^2^ | 0.08 | 0.08 | 0.08 | 0.08 | 0.08 | 0.08 | 0.08 |
| Observations | 10720 | 10720 | 10720 | 10720 | 10720 | 10720 | 10720 |

***Notes:*** *Outcome: Choosing the respective candidate to receive the vaccine. Coefficients are odds ratios based on conditional logit estimations (respondent-level fixed effects) with standard errors clustered at the respondent level. Estimations were conducted with controlling for the main effects of the other three attributes, but only the results for the country of residence attribute are shown here. Columns 2-7 indicate the degree of statistical (in-)significance of the subgroup differences presented in Figure 2. Results to be interpreted relative to the indicated reference category, i.e. in the case of country of residence, relative to the preference for the vaccine being given to a person living in the country of the survey respondent answering the question. 95% confidence intervals in brackets. ^∗^ p < 0.05, ^∗∗^ p < 0.01, ^∗∗∗^p < 0.001.*

**Supplementary File 1i** – Country of residence attribute: Heterogeneity by respondent’s characteristics

| Global South | 1.43^∗∗∗^ | 1.27^∗^ | 1.78^∗∗∗^ | 1.39^∗∗^ | 1.60^∗∗∗^ | 1.38^∗∗^ | 1.63^∗∗∗^ |
| --- | --- | --- | --- | --- | --- | --- | --- |
|  | [1.24,1.65] | [1.02,1.56] | [1.52,2.09] | [1.09,1.76] | [1.37,1.88] | [1.13,1.68] | [1.22,2.17] |
| Global South × Female respondent |  | 1.28 |  |  |  |  |  |
|  |  | [0.98,1.67] |  |  |  |  |  |
| Global South × Respondent ≥ 45 |  |  | 0.48^∗∗∗^ [0.35,0.66] |  |  |  |  |
| Global South × Higher educated respondent |  |  |  | 1.05 |  |  |  |
|  |  |  |  | [0.79,1.39] |  |  |  |
| Global South × High-risk respondent |  |  |  |  | 0.57^∗∗^ [0.41,0.80] |  |  |
| Global South × High perceived threat respondent |  |  |  |  |  | 1.07 |  |
|  |  |  |  |  |  | [0.82,1.40] |  |
| Global South × Employed respondent |  |  |  |  |  |  | 0.85 |
|  |  |  |  |  |  |  | [0.61,1.18] |
| Pseudo R^2^ | 0.23 | 0.23 | 0.23 | 0.23 | 0.23 | 0.23 | 0.23 |
| Observations | 15008 | 15008 | 15008 | 15008 | 15008 | 15008 | 15008 |

(Swedish sample)

|  | (1) | (2) | (3) | (4) | (5) | (6) | (7) |
| --- | --- | --- | --- | --- | --- | --- | --- |
| ***Country of Residence*** |  |  |  |  |  |  |  |
| Respondents’ Country | Reference Category | | | | | | |

***Notes:*** *Outcome: Choosing the respective candidate to receive the vaccine.* *Coefficients are odds ratios based on conditional logit estimations (respondent-level fixed effects) with standard errors clustered at the respondent level. Estimations were conducted with controlling for the main effects of the other three attributes, but only the results for the country of residence attribute are shown here. Columns 2-7 indicate the degree of statistical (in-)significance of the subgroup differences presented in Figure 2. Results to be interpreted relative to the indicated reference category, i.e. in the case of country of residence, relative to the preference for the vaccine being given to a person living in the country of the survey respondent answering the question. 95% confidence intervals in brackets. ^∗^ p < 0.05, ^∗∗^ p < 0.01, ^∗∗∗^p < 0.001.*

**Supplementary File 1j -** Country of residence attribute main effect - By subgroups of regional case incidence (pooled sample)

|  |  |  | **Self-constructed intervals** of regional incidence rate (cases/100.000) | | | | |  | | **Quintiles** of regional incidence rate (cases/100.000) | | | | | |
| --- | --- | --- | --- | --- | --- | --- | --- | --- | --- | --- | --- | --- | --- | --- | --- |
|  | (1) |  | (2) | (3) | (4) | (5) | (6) |  | | (7) | (8) | (9) | (10) | (11) |  |
|  | Full sample |  | <50 | 50-99 | 100-249 | 250-349 | ≥350 |  | | <40 | 40-50 | 51-90 | 91-322 | ≥322 |  |
| **Country of residence** |  |  |  |  |  |  |  |  |  |  |  |  |  |  |  |
| Respondents’ country |  |  | Reference category | | | | | | Reference category | | | | | | |
| Global South | 1.16∗∗∗  [1.10,1.23] |  | 1.26∗∗∗ [1.16,1.38] | 1.60∗∗∗  [1.44,1.78] | 1.46∗∗∗  [1.23,1.41] | 0.72∗∗∗  [0.64,0.80] | 0.60∗∗  [0.45,0.82] |  | | 1.17∗∗  [1.05,1.30] | 1.49∗∗∗  [1.31,1.70] | 1.59∗∗∗  [0.41,1.79] | 1.08  [0.95,1.23] | 0.69∗∗∗  [0.60,0.78] |  |
| Pseudo *R*^2^ | 0.16 |  | 0.14 | 0.17 | 0.17 | 0.22 | 0.19 |  | | 0.11 | 0.18 | 0.17 | 0.18 | 0.22 |  |
| Observations | 96480 |  | 34320 | 23936 | 10160 | 24896 | 3168 |  | | 21792 | 16768 | 19424 | 18992 | 19120 |  |

***Notes*:** *Outcome: Choosing the respective candidate to receive the vaccine. Coefficients are odds ratios based on conditional logit estimations (respondent-level fixed effects) with standard errors clustered at the respondent level. Estimations were conducted with all four attributes, but only the results for the country of residence attribute are shown here. Data for the regional COVID-19 case incidence was drawn from <https://www.ecdc.europa.eu/en/publications-data/weekly-subnational-14-day-notification-rate-covid-19>. Columns 2-11 represent the exact coefficients shown in Figure5 in the main manuscript. Coefficients are to be interpreted relative to the indicated reference category, i.e. in the case of country of residence, relative to the preference for the vaccine being given to a person living in the country of the survey respondent answering the question. 95% confidence intervals in brackets. ∗ p < 0.05, ∗∗ p < 0.01, ∗∗∗ p < 0.001****.***

**Supplementary File 1k** – Country of residence attribute: Heterogeneity by regional case incidence

(pooled results)

|  | |  | **Continuous** **coding**  of case incidence | **Categorical coding**  of case incidence  *(Self-constructed intervals)* | **Categorical coding**  of case incidence  *(Quintiles of distribution)* |
| --- | --- | --- | --- | --- | --- |
|  | | (1) | (2) | (3) | (4) |
| ***Country of Residence*** | |  |  |  |  |
| Respondents’ Country | Reference category | | Reference category | Reference category | Reference category |
| Global South | | 1.16∗∗∗  [1.10,1.23] | 1.608∗∗∗  [1.49,1.74] | 1.29∗∗∗  [1.18,1.41] | 1.22∗∗∗  [1.09,1.36] |
| Global South × Incidence | |  | 0.998∗∗∗  [0.997,0.998] |  |  |
| Global South × Incidence <50 | |  |  | Reference category |  |
| Global South × Incidence 50-99 | |  |  | 1.30∗∗∗  [1.14,1.49] |  |
| Global South × Incidence 100-249 | |  |  | 1.11  [0.92,1.32] |  |
| Global South × Incidence 250-349 | |  |  | 0.56∗∗∗  [0.49,0.64] |  |
| Global South × Incidence ≥350 | |  |  | 0.45∗∗∗  [0.36,0.61] |  |
| Global South × Incidence <40 | |  |  |  | Reference category |
| Global South × Incidence 40-50 | |  |  |  | 1.18∗  [1.00,1.39] |
| Global South × Incidence 51-90 | |  |  |  | 1.38∗∗∗  [1.18,1.61] |
| Global South × Incidence 91-322 | |  |  |  | 0.84∗  [0.71,0.98] |
| Global South × Incidence ≥322 | |  |  |  | 0.58∗∗∗  [0.50,0.69] |
| Pseudo R^2^ | | 0.16 | 0.17 | 0.17 | 0.17 |
| Observations | | 96480 | 96096 | 96096 | 96096 |

***Notes:*** *Outcome: Choosing the respective candidate to receive the vaccine.* *Coefficients are odds ratios based on conditional logit estimations (respondent-level fixed effects) with standard errors clustered at the respondent level. Estimations were conducted with controlling for the main effects of the other three attributes, but only the results for the country of residence attribute are shown here. Data for the regional COVID-19 case incidence was drawn from <https://www.ecdc.europa.eu/en/publications-data/weekly-subnational-14-day-notification-rate-covid-19>. Column 3 indicates the degree of statistical (in-)significance of the subgroup differences presented in Figure 5 in the main body of the paper. Results to be interpreted relative to the indicated reference category, i.e. in the case of country of residence, relative to the preference for the vaccine being given to a person living in the country of the survey respondent answering the question. 95% confidence intervals in brackets. ^∗^ p < 0.05, ^∗∗^ p < 0.01, ^∗∗∗^p < 0.001.*

**Supplementary File 1l** – Country level differences in case incidence, vaccination rates, willingness and threat perception

|  | Germany | Spain | Italy | France | Poland | Sweden |
| --- | --- | --- | --- | --- | --- | --- |
| **Time of data collection** | 9.-30.4.21 | 15.-21.6.21 | 15.-21.6.21 | 15.-21.6.21 | 15.-21.6.21 | 15.-24.6.21 |
| **Reported COVID-19 cases/100.000 people** | 315.65 | 97.65 | 47.26 | 51.50 | 7.80 | 59.54 |
| **Vaccination rate (first shot)** | 25.3% | 59.9% | 62.3% | 60.2% | 52.5% | 56.8% |
| **Vaccination rate (both shots)** | 8.0% | 36.5% | 31.4% | 30.9% | 36.3% | 34.2% |
| **Vaccination willingness** (control) | 2.14 | 2.63 | 2.18 | 1.77 | 1.68 | 2.28 |
| (1=unsure; 2=depends on vaccine; 3=sure) | (0.69) | (0.68) | (0.77) | (0.73) | (0.73) | (0.81) |
| **Vaccination willingness** (full sample) | 2.22 | 2.59 | 2.15 | 1.79 | 1.71 | 2.29 |
| (1=unsure; 2=depends on vaccine; 3=sure) | (0.70) | (0.69) | (0.76) | (0.76) | (0.76) | (0.80) |
| **COVID-19 threat perception** (initial coding) | 4.57 | 3.52 | 3.71 | 3.36 | 2.97 | 3.39 |
| (Germany: 7-point scale; Other countries: 5-point scale) | (1.67) | (1.12) | (1.06) | (1.10) | (1.20) | (1.08) |
| **Elevated COVID-19 threat perception** | 0.54 | 0.53 | 0.58 | 0.46 | 0.33 | 0.49 |
| (dummy coded, Germany: 1-4=0; 5-7=1; Other countries: 1-3=0; 4-5=1) | (0.50) | (0.50) | (0.49) | (0.50) | (0.47) | (0.50) |

***Notes:*** *The vaccination rates reported here are the averages for the time during which each survey was in the field. Data was drawn from* [*https://vaccinetracker.ecdc.*](https://vaccinetracker.ecdc.europa.eu/public/extensions/COVID-19/vaccine-tracker.html) [*europa.eu/public/extensions/COVID-19/vaccine-tracker.html.*](https://vaccinetracker.ecdc.europa.eu/public/extensions/COVID-19/vaccine-tracker.html) *The case incidence rates reported here represent the 14-day notification rate of reported COVID-19 cases per 100 000 people, averaged over the data collection time period and sampled regions of the respective country. Data was drawn from https://www.ecdc.europa.eu/en/publications-data/weekly-subnational-14-day-notification-rate-covid-19. The vaccine willingness results were calculated both for the full sample and for a smaller subsample. The latter served as the control group in another survey experiment conducted throughout this same data collection, in which participants received different messages intended to reduce vaccine hesitancy. Thus, to make sure the vaccine willingness results are unaffected by this, we additionally report values from the control group.*
